# Effectiveness of Chêneau brace treatment for idiopathic scoliosis: prospective study in 79 patients followed to skeletal maturity

**DOI:** 10.1186/1748-7161-7-S1-O44

**Published:** 2012-01-27

**Authors:** I Kowalski, K Zaborowska-Sapeta, T Giżewski, T Kotwicki, H Protaziewicz-Fałdowska, W Kiebzak

**Affiliations:** 1 Department of Rehabilitation, Faculty of Medical Sciences, University of Warmia and Mazury, Olsztyn, Poland; 2 Institute of Electrical Engineering and Electrotechnologies, Faculty of Electrical Engineering and Computer Science, University of Technology, Lublin, Poland; 3 Department of Pediatric Orthopedics and Traumatology, University of Medical Sciences, Poznan, Poland; 4 Institute for Physiotherapy, Faculty of Medical Sciences, University of Kielce, Poland

## Background

Progressive idiopathic scoliosis can influence negatively the development and function of 2-3% of adolescents, with health consequences and economic costs, placing the disease in the centre of interest of medicine of the growing age. The aim of this study was to evaluate the effectiveness of Chêneau brace in the management of idiopathic scoliosis.

## Materials and methods

A prospective observational study according to SOSORT and SRS recommendations comprised 79 patients (58 girls and 21 boys) with progressiveidiopathic scoliosis, treated with Chêneau brace and physiotherapy, with initial Cobb angle between 20 and 45 degrees, no previous brace treatment, Risser 4 or more at the final evaluation and minimum one year follow-up after weaning the brace. Achieving 50° of Cobb angle was considered surgical recommendation.

## Results

At follow-up 20 patients (25.3%) improved, 18 patients (22.8%) were stable, 31 patients (39.2%) progressed below 50 degrees and 10 patients (12.7%) progressed beyond 50 degrees (2 of these 10 patients progressed beyond 60 degrees). Progression concerned the younger and less skeletally mature patients.

## Conclusions

Conservative treatment with Chêneau orthosis and physiotherapy was effective in halting scoliosis progression in 48.1% of patients. The results of this study suggest that bracing is effective in reducing the incidence of surgery in comparison with natural history.

